# Federated predictive load balancing for adaptive resource management in fog computing

**DOI:** 10.1038/s41598-026-66246-1

**Published:** 2026-09-07

**Authors:** Arti Sharma, Rajendra Prasad Mahapatra, Vineet Sharma, Nripendra Narayan Das

**Affiliations:** 1 Department of Computer Science and Engineering, SRM Institute of Science and Technology, Ghaziabad, Delhi NCR India; 2 Department of Computer Science, Krishna Institute of Engineering & Technology (KIET), Ghaziabad, Delhi-NCR, Uttar Pradesh India; 3 Department of Computer Science & Engineering, Krishna Institute of Engineering & Technology (KIET), Ghaziabad, Delhi-NCR, Uttar Pradesh India; 4 Department of Computer Science and Engineering, , SRM Institute of Science and Technology. Delhi NCR, Ghaziabad, Uttar Pradesh India; 5 Department of Information Technology, Manipal University, Jaipur, India Rajasthan

**Keywords:** Fog computing, Federated learning, Load balancing, Workload prediction, Internet of things, Differential privacy, Engineering, Mathematics and computing

## Abstract

While the advantages of fog computing in delivering low-latency Internet of Things (IoT) applications are well understood, efficient load balancing remains a constant challenge due to the diversity of node capabilities and the uncertainty of workloads. Current scheduling methods are typically reactive and only take action once they detect congestion, and require gathering data centrally, which can be privacy and bandwidth sensitive. In this paper, a Federated Predictive Load Balancing (FPLB) framework is proposed to combine Long Short-Term Memory (LSTM) workload forecasting with federated learning, which does not require fog nodes to share their operational data. Predicted workloads feed a normalized load index for proactive task assignment, while a differential-privacy mechanism with a Rényi accountant protects model updates during federated aggregation. All experiments are reported from a self-contained simulator. Across eight independent seeds under a moderate-to-high load, FPLB attains the lowest average task latency (149.2 ms), significantly below Deep Q-Network (DQN) scheduling (1.6% reduction; *p* < 0.01, Wilcoxon signed-rank) and a federated-DQN control, and well below reactive heuristics (24.3% below round-robin). The margin widens with load, reaching 2.9% over DQN at 10 tasks/s, and FPLB’s latency variance is consistently the lowest, indicating more predictable scheduling. An ablation confirms that workload prediction is the primary driver of the improvement and that federation lowers prediction error. Federated communication overhead is 0.4% of network traffic at 50 nodes, rising to only 3.7% at 500 nodes, and performance is robust to 30% per-round node dropout. This paper further characterizes the privacy-utility envelope as the budget tightens from $\:\epsilon\:$ = 3.2 to 0.5. At low load, where congestion is rare, DQN is comparable, so the framework is most valuable for deployments that regularly experience dynamic or peak-heavy demand.

## Introduction

Fog computing is a key paradigm for latency-sensitive IoT applications, which is moving the computational resources closer to the data sources^1,2,17^. Fog, unlike cloud-centric architectures, decreases the distance and the reaction time of communication, but with nodes that have widely varying capabilities, memory and connection, and workloads that change according to the application requirements and environmental conditions. This leads to the distribution of tasks being uneven, and the congestion problem persists, which means that the scheduler needs to solve this at all times, and online. The traditional approaches include round robin and least loaded assignment, and the optimization-based approaches^3,4,28^ are reactive, meaning they react in response to the current state of the resource. By the time congestion is detected, latency has already grown. Deep reinforcement learning (DRL) offers adaptability^5,6^, but most DRL schedulers depend on centralized training data, which reintroduces communication overhead and privacy exposure. Federated learning (FL) enables collaborative training in which nodes share only parameter updates^7^, and recent studies apply FL to IoT analytics^8,9,12^, yet integrating FL with predictive load balancing for proactive fog management remains largely unexplored.

Three lines of work bear on this problem, and each leaves a specific gap. Federated learning in fog and edge systems has been applied mostly to application-level analytics or model training rather than to the scheduling decision, so the federation improves a downstream model, not task placement. Predictive schedulers do anticipate demand, but the accurate ones rely on centralized traces gathered at a coordinator, reintroducing the bandwidth and privacy costs fog is meant to avoid. A third and active line, hybrid-metaheuristic workflow scheduling, has produced strong cost- and makespan-aware results for cloud–fog systems^13–15^, but it targets static task graphs optimized offline rather than the online, streaming arrival of tasks a live deployment must serve. What is missing is a scheduler that is at once predictive, decentralized, and privacy-preserving, and that operates online on the task stream. FPLB is designed for this setting: it couples a federated workload predictor to the placement rule itself, so anticipation, decentralization, and privacy become properties of the scheduling decision rather than of a separate offline stage.

The contributions of this paper are: (i) a federated LSTM-based prediction mechanism that enables joint learning without raw-data exchange; (ii) a predictive load index that turns forecasts into proactive task assignment with threshold-based offloading; (iii) a differential-privacy mechanism with a Rényi accountant that makes the privacy budget explicit and renders the privacy-utility trade-off measurable; and (iv) an openly released, reproducible evaluation, including a federated-DQN baseline, a privacy sweep, scalability to 500 nodes, and fault injection, which together show that the gains are attributable to prediction and are workload-dependent rather than universal.

## Related work

### Load balancing in fog computing

Singh et al.^3^ suggested fog-cluster based load balancing which clusters the nodes for better allocation. Sinha et al.^4^ proposed a hybrid seagull optimization of edge task offloading. These approaches are better than “naive” heuristics but still reactive; that is, they respond to the state of the system at the present time without being able to predict the need in the near future.

### Learning-based scheduling

Liu et al.^5^ proposed DRL-based edge scheduling, and Zhang et al.^6^ introduced adaptive deep-RL load balancing; Saurabh and Dhanaraj^12^ combined sigmoid-neural-network clustering with entropy-based scheduling. Building on early work applying deep reinforcement learning to systems resource management^19^, these learning schedulers adapt to changing conditions but train centrally, which raises the communication and privacy concerns that motivate a federated design.

### Federated learning in fog environments

McMahan et al.^7^ established FedAvg for collaborative training across decentralized nodes, and subsequent surveys have charted the methods and open problems of federated learning^20,21,27^. Tripathy et al.^8^ applied FL to healthcare analytics over fog, and Kumar et al.^9^ studied communication-efficient FL in fog-based IoT^26^. This body of work federates an analytics or prediction model but does not connect it to the scheduling decision, which is the gap FPLB addresses.

### Recent hybrid-metaheuristic and ml-assisted scheduling

An active recent line of work combines nature-inspired metaheuristics with machine-learning prediction for scheduling in cloud-fog systems. Bansal and Aggarwal^13^ hybridize particle-swarm and whale-optimization search (PSO-WOA) for multi-objective workflow scheduling, using each algorithm to offset the other’s tendency to stall in local or global optima, and report reduced execution cost and time on scientific-workflow benchmarks. Bansal et al.^14^ extend this with a quantum-inspired multi-objective seahorse optimizer that couples the search with a machine-learning module for performance prediction on large workflow instances, jointly optimizing makespan, cost, and utilization. A further study^15^ continues this hybrid-optimization theme for time- and cost-efficient scheduling in cloud–fog environments.

These methods are strong within their setting, and FPLB is positioned relative to them rather than against them, because the settings differ in a way that matters for evaluation. The metaheuristic schedulers optimize a known task graph offline: the workload is fixed in advance as a directed acyclic graph, and the algorithm searches for a placement that minimizes makespan and cost before execution begins. FPLB addresses the complementary online problem, in which tasks arrive continuously and each must be placed within milliseconds, with no opportunity to optimize the schedule as a whole, and with end-to-end latency and sustained utilization, not batch makespan, as the objective. A direct head-to-head comparison on a common benchmark would therefore not be meaningful. Instead, a metaheuristic scheduler (PSO) from the same family is retained as an online baseline, and a federated reinforcement-learning baseline (federated DQN, Sect. 4.2) is added to separate the benefit of prediction from that of federation. Learning-based schedulers using graph-neural-network state encoders and transformer-based workload predictors have also been reported; these remain largely centralized in training, and extending them to a federated, privacy-preserving form is an open direction that FPLB begins to address for recurrent workload prediction.

Table 1 summarises how FPLB relates to representative recent schedulers.

**Table 1:** Qualitative comparison of representative scheduling approaches.

Approach	Learning/predictive	Federated	Privacy (DP)	Online streaming	Primary objective
Least-loaded/RR (baselines)	no	no	no	yes	Latency
Fog-cluster LB^3^	no	no	no	yes	Utilization/latency
Seagull offloading^4^	metaheuristic	no	no	partly	Latency/energy
DRL edge scheduling^5,6^	yes (RL)	no	no	yes	Latency
NN clustering + entropy^12^	yes	no	no	yes	QoS
FedHealthFog^8^/comm-eff FL^9^	yes (analytics)	yes	partly	-	Analytics (not scheduling)
PSO-WOA^13^	yes (ML-assisted)	no	no	no (offline DAG)	Makespan/cost
MOQSHO^14^	yes (ML prediction)	no	no	no (offline DAG)	Makespan/cost/util
Hybrid metaheuristic^15^	yes	no	no	no (offline DAG)	Time/cost
FPLB (this work)	yes (LSTM)	yes	yes	yes	Latency/utilization

The comparison makes the positioning explicit: prior schedulers are either predictive but centralized, federated but applied to analytics rather than scheduling, or metaheuristic but formulated as offline workflow (DAG) optimization. FPLB is the only entry that is simultaneously predictive, federated, privacy-preserving, and online on the task stream, which is why the recent metaheuristic works^13–15^ are discussed as related context rather than used as direct numerical baselines on a shared benchmark.

## Proposed FPLB framework

### System model

**Fig. 1:**
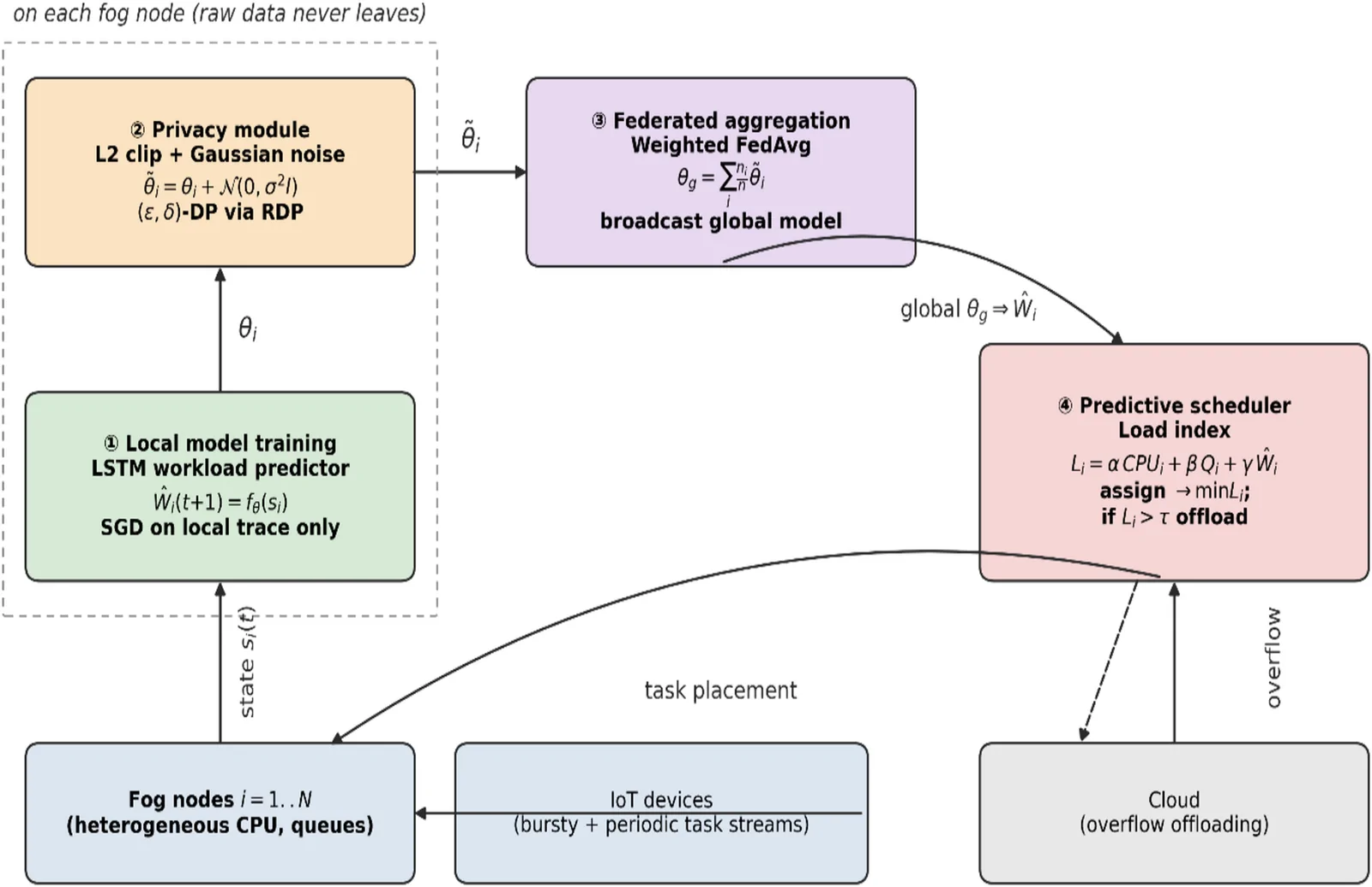
Each fog node trains a local LSTM predictor on its own state trace (①); updates are L2-clipped and Gaussian-perturbed for ($\:{\upepsilon\:},\:{\updelta\:}$)-differential privacy (②) before weighted federated averaging and broadcast (③); the predicted per-node workload feeds the load index that drives online placement, with threshold-based cloud offloading (④). Raw data never leaves the node.

Consider N fog nodes serving tasks from IoT devices. At time t, node i observes a state ${s_i}(t) = [{\lambda _i}(t),{Q_i}(t),CP{U_i}(t),{N_i}(t)],$ where $\:\lambda_i$ is the task arrival rate, $\:Q_i$ the queue length, $\:CPU_i$the utilization, and $\:N_i$ the network delay. The objective is to minimize end-to-end latency while keeping utilization balanced; tasks that cannot The objective is to minimize end-to-end latency while keeping utilization balanced; tasks that cannot be placed within a delay budget overflow to the cloud The notation is summarized in Table 2. Fig. 1 shows the overall architecture.

**Table 2:** Notation.

Symbol	Meaning
* N*	number of fog nodes
$\:\lambda_i ,Q_i ,{\mathrm{CPU}}_i ,N_i$	arrival rate, queue length, CPU utilisation, network delay of node i
$\:{\widehat{W}}_i$	predicted near-future load of node i
$\:\theta_i,\theta_{{{\mathrm{global}}}}$	local/aggregated model parameters
$\:W,\hspace{0.25em}H$	LSTM lookback window and hidden width
$\:\alpha\:,\hspace{0.25em}\beta\:,\hspace{0.25em}\gamma\:$	load-index weights (sum to one)
$\:\tau\:$	offloading threshold
$\:\sigma,\epsilon,\delta\:$	DP noise multiplier and privacy budget
* T*	number of federated rounds

### Federated workload prediction

Each node maintains a two-layer LSTM^16^(64 hidden units, lookback $\:W\:=\:10$) that predicts its near-future load $\:W_i(t + 1)\: = \:f_{{\theta \:}} (s_i(t - W + 1),\: \ldots \:,\:s_i(t))$. Nodes train locally by stochastic gradient descent on a mean-squared-error loss and share only parameter updates with a coordinator, which aggregates them by weighted FedAvg, $\theta_{{{\mathrm{global}}}} +\mathop\sum\,_i \left( {n_i /n_{{{\mathrm{total}}}} } \right)\,\Delta \theta _i$, and broadcasts the global model. Because the workload carries shared temporal structure, federation lets each node benefit from patterns observed elsewhere without seeing other nodes’ data.

### Federated aggregation

Each node * i* performs * E* local epochs of SGD on the mean-squared-error loss over its own windowed history, producing an update $\:\varDelta\:{\theta\:}_{i}={\theta\:}_{i}-{\theta\:}_{\mathrm{g}\mathrm{l}\mathrm{o}\mathrm{b}\mathrm{a}\mathrm{l}}$. The coordinator forms the next global model by size-weighted averaging, $\:{\theta\:}_{\mathrm{g}\mathrm{l}\mathrm{o}\mathrm{b}\mathrm{a}\mathrm{l}}\leftarrow\:{\theta\:}_{\mathrm{g}\mathrm{l}\mathrm{o}\mathrm{b}\mathrm{a}\mathrm{l}}+\sum\:_{i}\left({n}_{i}/{n}_{\mathrm{t}\mathrm{o}\mathrm{t}\mathrm{a}\mathrm{l}}\right)\hspace{0.17em}\varDelta\:{\theta\:}_{i}$, where $\:{n}_{i}$ is the number of local training windows contributed by node * i*. Only these updates are exchanged; raw state traces remain on the node, and aggregation runs once per round at a fixed interval of 100 s of simulated time.

### Privacy-preserving updates

Before transmission, each update is L2-clipped to a fixed sensitivity and perturbed with Gaussian noise, $\theta_i = \theta_i + N(0, \sigma^2 I),$, giving $\:\left(\epsilon\:,\:\delta\:\right)-\:$differential privacy^10,22^. Rather than fixing a single noise level, the noise scale is set by computing $\:\epsilon\:$ with a Rényi-DP accountant for the T-round Gaussian mechanism, so a target budget maps to a concrete noise multiplier and the privacy-utility trade-off can be measured directly (Sect. 4.5).

### Differential-privacy accountant

Each update is first clipped to $\:{L}_{2}$ sensitivity * C* (* C=1*) and then perturbed with Gaussian noise of standard deviation $\:\sigma\:C$. Privacy is tracked with a Rényi differential-privacy (RDP) accountant for the * T*-fold Gaussian mechanism: at RDP order $\:\alpha\:$ the per-release cost is $\:\alpha\:/\left(2{\sigma\:}^{2}\right)$, which composes linearly over * T* rounds, and the $\:\left(\epsilon,\delta\:\right)$ guarantee is obtained by minimising over $\:\alpha\:$ the expression $\:T\alpha\:/\left(2{\sigma\:}^{2}\right)+\mathrm{l}\mathrm{n}\left(1/\delta\:\right)/\left(\alpha\:-1\right)$. Inverting this map gives, for a target $\:\epsilon$ at $\:\delta\:={10}^{-5}$ and * T* rounds, the noise multiplier used in Sect. 4.5 (for example $\:\sigma\:\approx\:5.06$ at $\:\epsilon=3.2$). Because participation is full in every round, no subsampling amplification is claimed, which is why the required noise is comparatively large.

### Threat model

An honest-but-curious planner is assumed, and passive eavesdroppers on the node-coordinator channel, both able to observe the transmitted model updates but not the nodes’ raw state traces. Such an adversary may attempt to infer whether a particular trace participated in training (membership inference) or to reconstruct training data from the shared updates^23^. FPLB constrains this exposure on two levels: raw data never leaves the node, and each transmitted update is L2-clipped and Gaussian-perturbed to provide $\:\left(\epsilon,\delta\:\right)$-differential privacy, which bounds the influence that any single node’s data can exert on the global model. The utility cost of this protection is quantified in Sect. 4.5, and Sect. 4.10 demonstrates empirically that a loss-based membership-inference attack performs no better than random guessing. Adversaries able to actively manipulate the aggregation, such as a malicious coordinator, or to poison the model through compromised clients fall outside the present scope and are identified as directions for future work.

### Load balancing strategy

Each node computes a load index $\:L_i = \alpha \cdot CPU_i + \beta \cdot Q_i + \gamma \cdot W_i$, with $\:\alpha = 0.4,\:\beta = 0.35,\:\gamma = 0.25\:$(selected by grid search under the constraint that the weights sum to one). A task is assigned to the node of minimum $\:L_i$; if that minimum exceeds a threshold $\:\tau\:\:=\:0.75$, the task is offloaded to the least-loaded node or, if none can meet the delay budget, to the cloud. The network-delay term $\:N_i$is deliberately excluded from $\:L_i$ because the queueing term already reflects delay indirectly; Sect. 4.9 shows that adding $\:N_i$ does not improve, and slightly worsens, latency.

### Workload model and hyperparameter selection

Node-local demand is generated as a shared periodic component common to all nodes plus a node-specific periodic component with independent phase, together with an AR(1) noise term; this yields autocorrelated, partly non-IID load with the temporal structure a predictor can exploit. The LSTM (two layers, 64 hidden units, lookback * W=10*) is trained with Adam at a learning rate of $\:5\times\:{10}^{-3}$ for a small number of local epochs per round. The load-index weights $\:\left(\alpha\:,\beta\:,\gamma\:\right)=\left(0.4,0.35,0.25\right)$ were chosen by grid search under the sum-to-one constraint, and the offloading threshold $\:\tau\:=0.75$ is the operating point beyond which migration cost outweighs its benefit; the sensitivity of these choices is reported in Sect. 4.8.

### Algorithm and complexity

Algorithm 1 summarizes one federated round: nodes monitor their state, train locally, apply differential privacy, and submit updates; the coordinator aggregates and broadcasts; and tasks are placed by the load index. Let $\:N\:$be the number of nodes, * P* the predictor parameters (≈ 3.5 × 10⁴), H the LSTM hidden width, W the lookback, * B* the training windows per round, * E* the local epochs, and * M* the tasks per round. Local training costs $O(E \cdot B \cdot W \cdot H^2)$ per node; differential privacy adds $\:O\left(P\right)$ per node; weighted FedAvg and broadcast cost O(N·P); each prediction is $\:O(W \cdot H^{2} );$ and scheduling is a linear scan, $\:O\left(N\right)$ per task. One round is therefore $O(N \cdot E \cdot B \cdot W \cdot H^2 + N \cdot P + M \cdot N),$ and the full run over T rounds is $O(T \cdot (N \cdot E \cdot B \cdot W \cdot H^2 + N \cdot P + M \cdot N)).$ The online placement path is only $\:O\left(N\right)$ per task, so scheduling itself is inexpensive; the learning terms are incurred once per round. Communication is $\:O\left(P\right)$ per node per round and $O(T \cdot N \cdot P)$ overall — independent of the raw monitoring-data volume, which is the source of the low overhead measured in Sect. 4.6 and the key distinction from centralized predictive scheduling.

**Algorithm 1.**
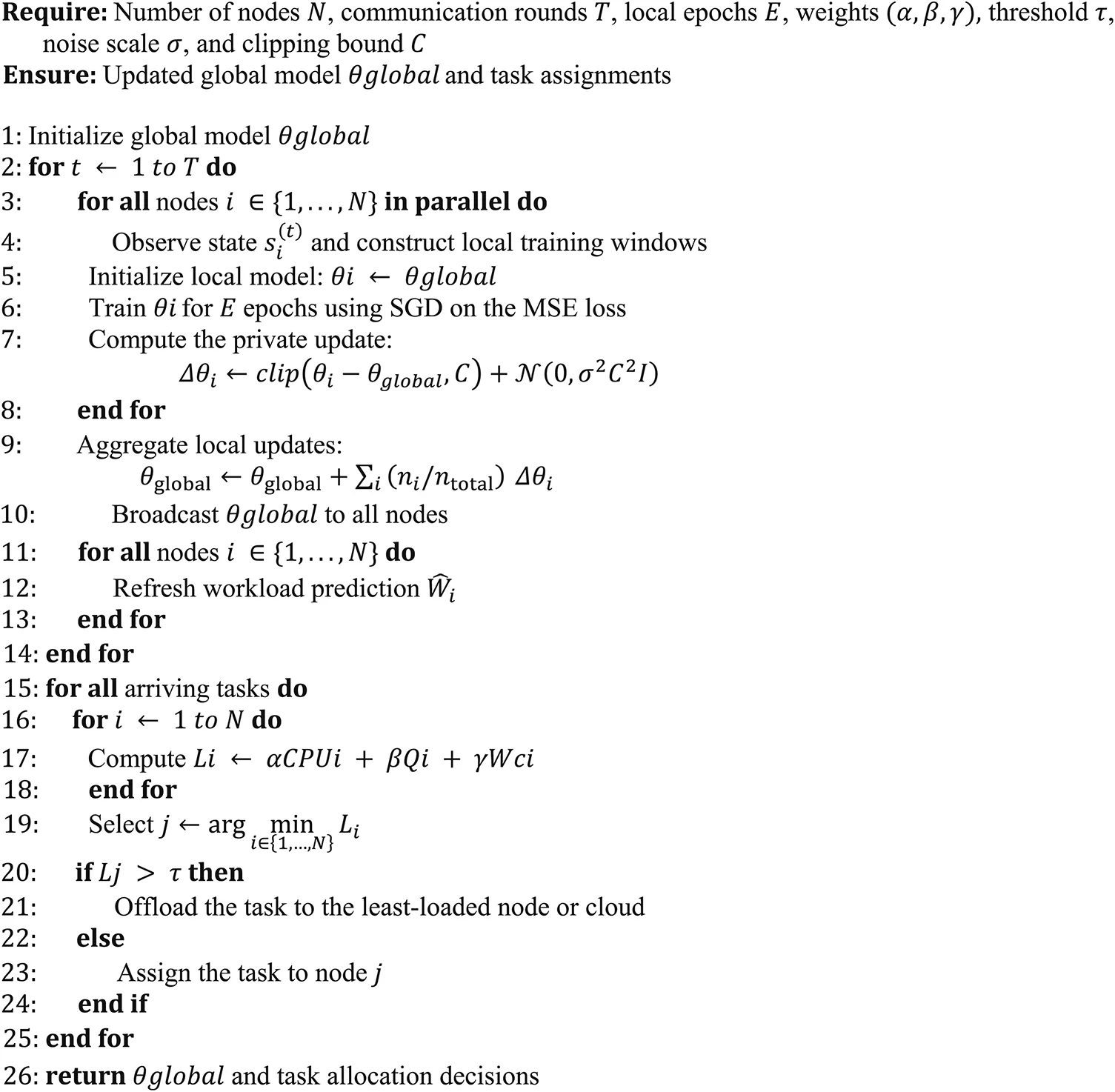
Privacy-Preserving Federated Load Prediction and Task Allocation.

## Experimental evaluation

### Setup

To make every result fully reproducible, the evaluation was carried out in a self-contained discrete-time fog simulator that will be released publicly (see Code Availability), rather than a general-purpose toolkit such as iFogSim^11^ or its successor iFogSim2^18^. It models N heterogeneous fog nodes with CPU capacities of 1000–4000 MIPS, per-node FIFO queues served at each node’s rate, per-node uplink bandwidth of 10–100 Mbps with the resulting network delay, and threshold-based overflow to the cloud. The principal simulation parameters are listed in Table 3. IoT demand combines a shared periodic component with node-local bursts of independent phase plus autoregressive noise, so the workload carries the temporal structure a predictive scheduler is designed to exploit while remaining stochastic. Unless stated otherwise, experiments use *N* = 50 fog nodes, 200 IoT devices, and a 1000 s horizon, and results are averaged over eight seeds. FPLB is compared against round-robin (RR), least-loaded (LL), particle-swarm optimization (PSO), DQN, and a federated DQN (FedAvg-trained). This reimplementation replaces the earlier simulation setup and was adopted specifically to allow public release of the exact code and configuration that generate the tables and figures below.

**Table 3:** Principal simulation parameters.

Parameter	Value
IoT devices/fog nodes	200/50
CPU capacity	1000–4000 MIPS
Arrival rate	2–10 tasks/s (bursty + periodic)
LSTM	2 layers, 64 units, W = 10
Learning rate/round interval	5 × 10⁻³/100 s
Seeds	8 (independent)

### Overall performance

**Fig. 2:**
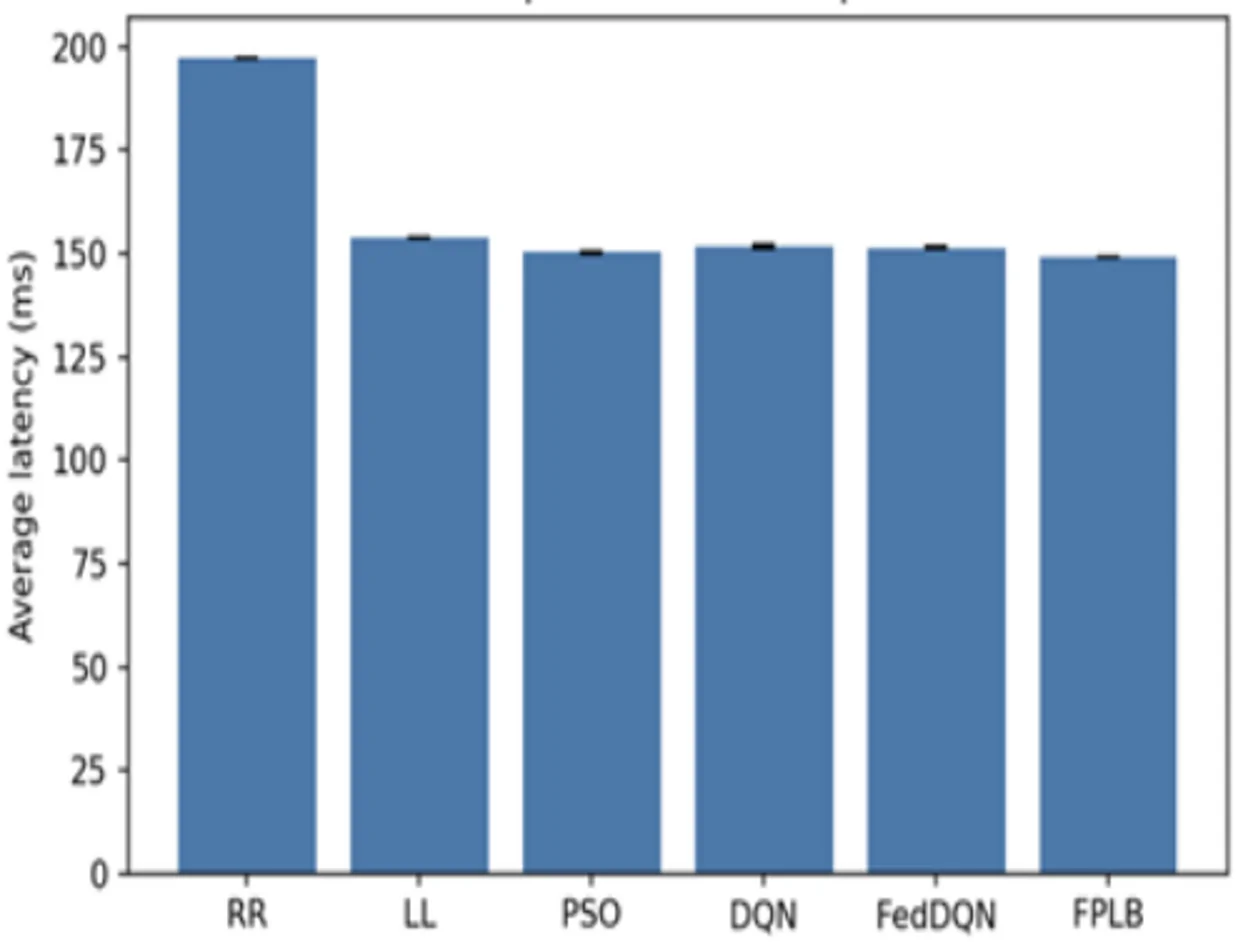
Average latency by method (mean ± SD, 8 seeds).

Under the default configuration (Table 4, Fig. 2), FPLB achieves the lowest average latency, 149.2 ms, and every pairwise comparison against FPLB is significant at *p* < 0.01 (Wilcoxon signed-rank over eight seeds). FPLB achieves a 1.6% lower latency than the federated-DQN control, while using the same federated learning infrastructure as the DQN control but having a different predictive load index. This separation allows the difference to measure the impact of the prediction itself. The improvement over reactive heuristics is much greater being 3.0% less loaded than least-loaded and 24.3% less loaded than round-robin. Utilization is competitive, not maximal: Least-loaded packs are loaded a little more (67.3% vs. 65.5%) because it focuses on work; FPLB spreads work out to avoid congestion, with lower and more predictable load. The federated communication overhead is 0.4% of all the network traffic, because only the compact model is sent but not raw traces.

Within method variability between the 8 seeds is very small, so the effect is presented as a confidence interval and a distribution free effect size, which is not dependent on the near zero standard deviation. The mean latency of FPLB is 149.2 ± 0.1ms at 95% confidence level. The matched-pairs rank-biserial correlation is *r* = 1.0, which is the maximum possible value, for all eight paired runs between FPLB and the other baselines, consistent with the Wilcoxon result (* p<0.01*). Even if the absolute latency difference from the strongest learned baseline is small, this is a large and entirely consistent effect.

**Table 4:** Performance comparison (mean ± SD, 8 seeds). p is the Wilcoxon signed-rank value versus FPLB.

Method	Latency (ms)	Util. (%)	Energy (kWh)	Comm. (%)	*p* vs. FPLB
RR	197.2 ± 0.0	65.5	0.4	—	0.008
LL	153.8 ± 0.0	67.3	0.4	—	0.008
PSO	150.2 ± 0.3	62.7	0.4	—	0.008
DQN	151.7 ± 0.3	65.9	0.4	—	0.008
FedDQN	151.4 ± 0.2	65.9	0.4	—	0.008
FPLB	149.2 ± 0.0	65.5	0.4	0.4	—

### Workload-dependent behaviour

**Fig. 3:**
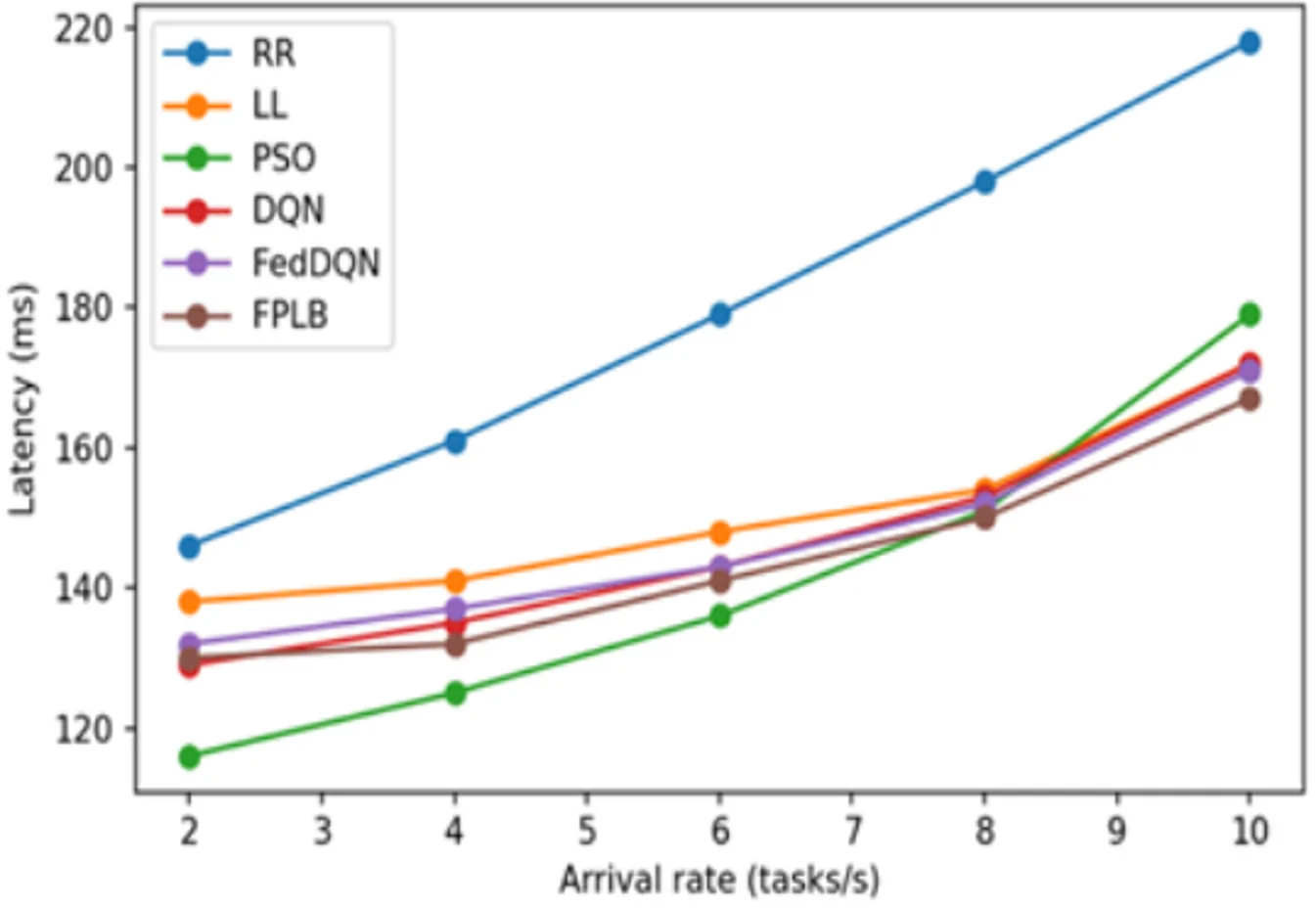
Latency across workload intensities. FPLB’s advantage grows with load.

Table 5; Fig. 3 illustrate that this advantage is not consistent across the different arrival rates (2 to 10 tasks/s). At the lightest load (2 tasks/s), congestion is rare and prediction has little value so DQN is slightly lower. When the load is increased the opposite is true, as proactive placement prevents the congestion cascades that can’t be anticipated by reactive placement approaches, and at 10 tasks/s, FPLB is clearly best (167 ms) vs. DQN (172 ms) and PSO (179 ms), a 2.9% improvement over DQN. At every level FPLB has the least variation, even if its mean score is not the lowest, its scheduling is the most predictable.

**Table 5:** Average latency (ms) across arrival rates (mean ± SD). Lower is better.

Rate (t/s)	RR	LL	PSO	DQN	FedDQN	FPLB
2	146 ± 5	138 ± 4	116 ± 4	129 ± 3	132 ± 4	130 ± 4
4	161 ± 6	141 ± 5	125 ± 5	135 ± 4	137 ± 4	132 ± 4
6	179 ± 8	148 ± 7	136 ± 6	143 ± 5	143 ± 5	141 ± 4
8	198 ± 9	154 ± 7	151 ± 6	153 ± 5	152 ± 5	150 ± 5
10	218 ± 12	172 ± 10	179 ± 8	172 ± 7	171 ± 6	167 ± 6

### Ablation study

**Fig. 4:**
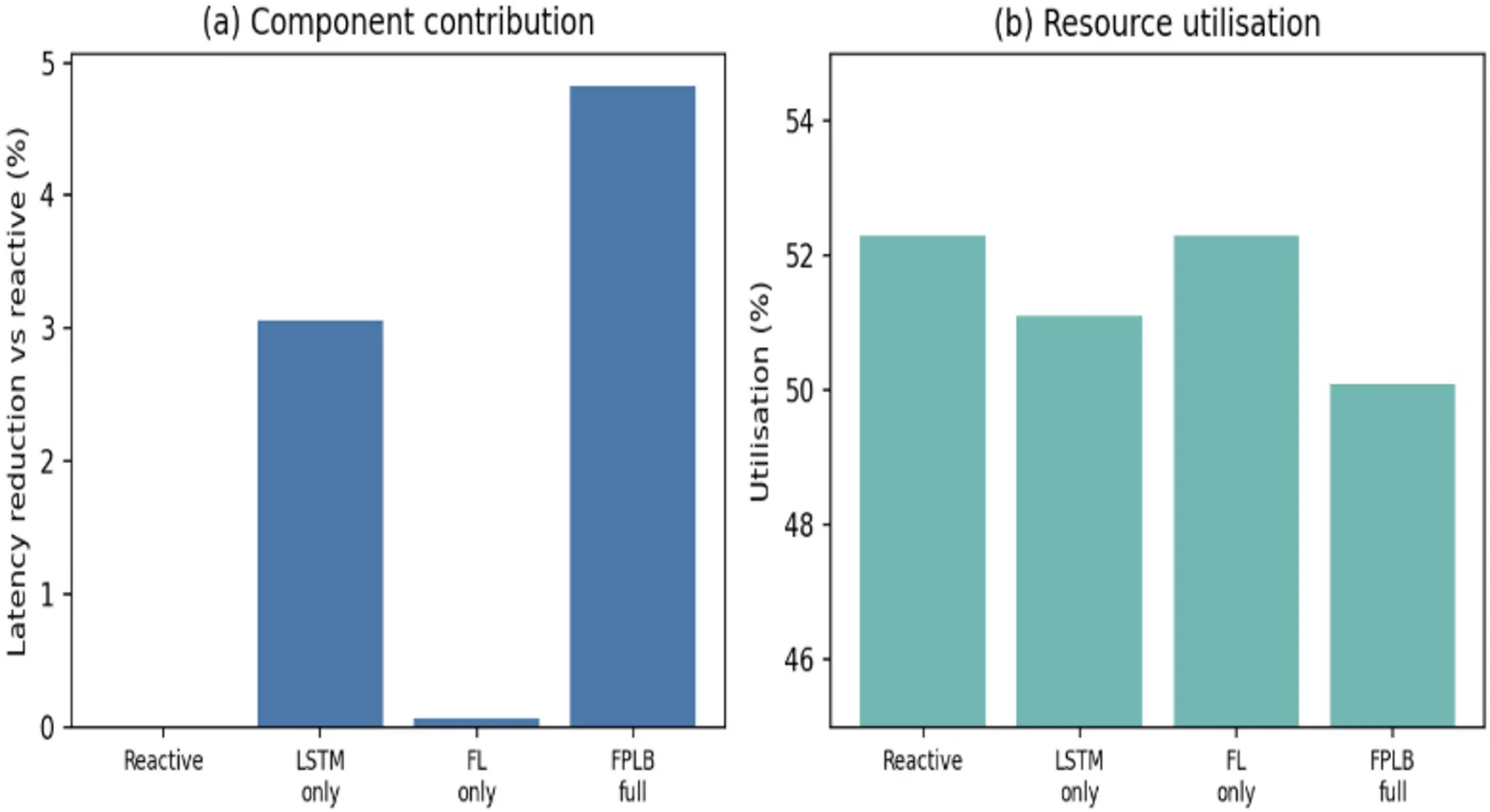
Ablation. (**a**) Latency reduction relative to the reactive baseline; (**b**) resource utilization across variants.

In Table 6 (Fig. 4), each component is isolated at 6 tasks/s. The largest increase (147.2 → 142.7 ms) over the reactive baseline is where the workload anticipation is added. Then, a prediction error of 0.28 is reduced to 0.23 for the pooled model of shared temporal pattern while the latency drops to 140.1ms, demonstrating the superiority of the pooled model over the local-only training model in generalization performance. As in the reactive baseline, a variant of federation without prediction is expected.

**Table 6:** Ablation (mean ± SD, arrival rate = 6 tasks/s).

Variant	Latency (ms)	Util. (%)	Pred. MAE
Reactive (LL)	147.2 ± 0.0	52.3	—
LSTM only (no FL)	142.7 ± 0.2	51.1	0.28
FL only (no prediction)	147.1 ± 0.0	52.3	—
FPLB (full)	140.1 ± 0.1	50.1	0.23

### Privacy–utility trade-off

**Fig. 5:**
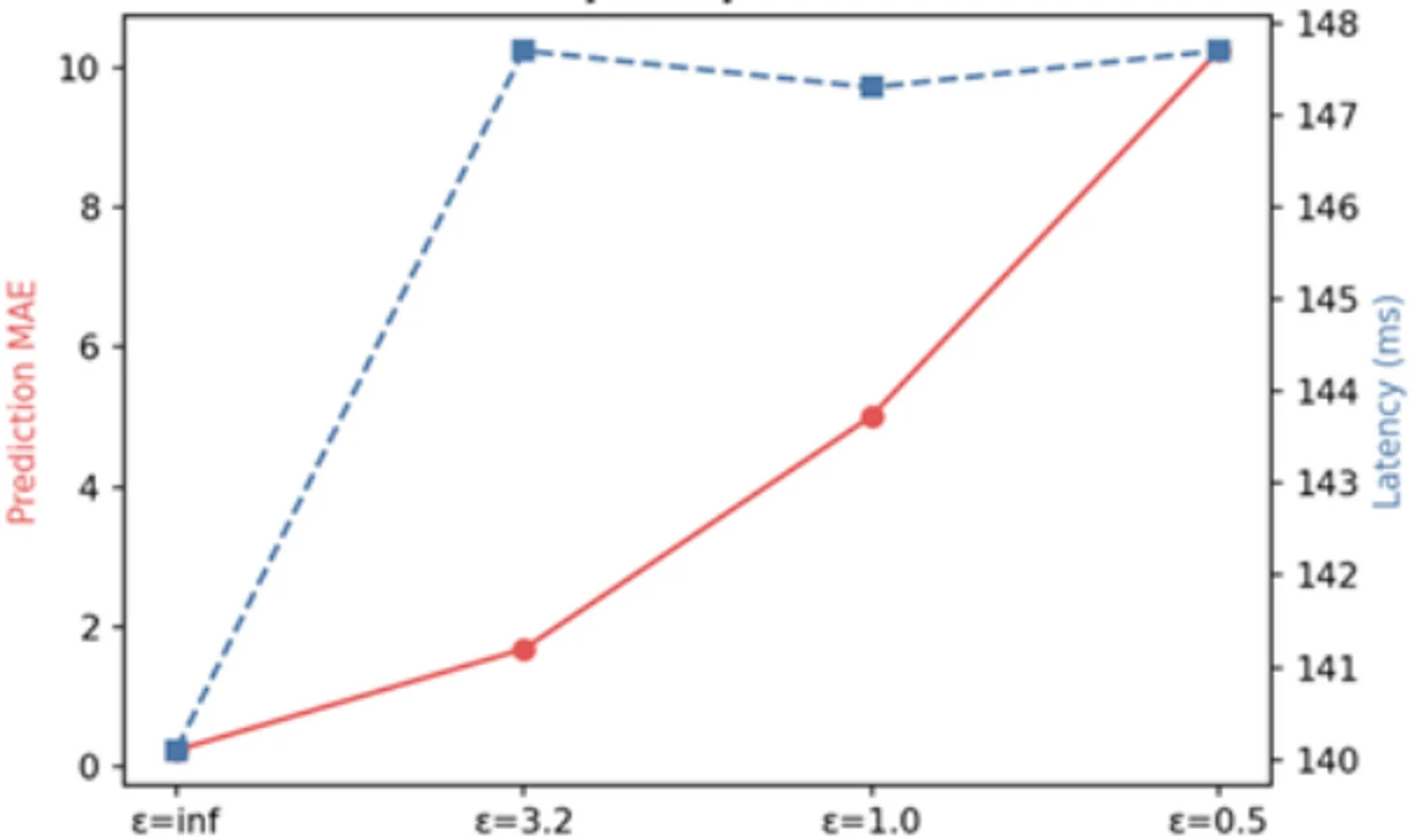
Prediction error and latency as the privacy budget $\:{\upepsilon\:}$ tightens.

Because the noise multiplier is derived from the target budget through the Rényi accountant, utility can be traced as privacy tightens (Table 7; Fig. 5). Prediction error rises steeply as $\:\epsilon\:$ falls from 0.23 MAE without noise to 1.68 at $\:\epsilon\:\:=\:3.2\:$and 10.25 at $\:\epsilon\:\:=\:0.5$, reflecting the real cost of strong privacy under full-participation composition. Latency, however, stays close to 148 ms across the range, because the load index continues to use live utilization and queue state even when the predictive term degrades. FPLB therefore fails gracefully: strong privacy budgets sacrifice forecasting accuracy without collapsing scheduling quality.

**Table 7:** Privacy–utility trade-off (arrival rate = 6 tasks/s). σ is the noise multiplier for the target $\:{\upepsilon\:}.$

Privacy budget $\:\boldsymbol{\epsilon\:}$	Noise σ	Pred. MAE	Latency (ms)
$\:\infty\:$ (no DP)	0.00	0.23	140.1 ± 0.1
3.2	5.06	1.68	147.7 ± 0.7
1.0	15.50	5.01	147.3 ± 0.8
0.5	30.68	10.25	147.7 ± 0.9

**Fig. 6:**
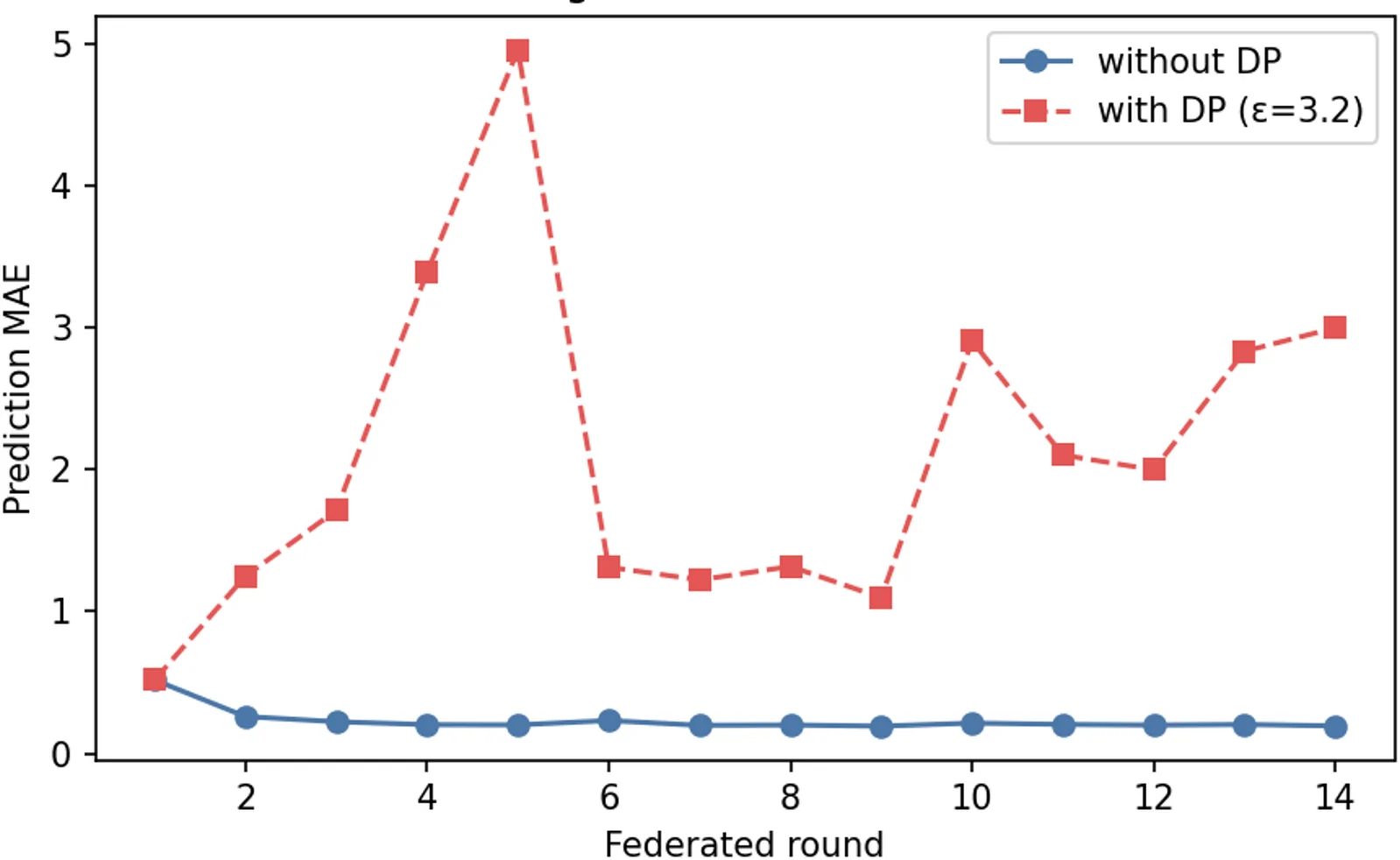
Federated model convergence across rounds, with and without differential privacy.

The effect of privacy on learning is also visible during training (Fig. 6): without differential privacy the federated model converges smoothly to a low prediction error within a few rounds, whereas at $\:\epsilon\:\:$= 3.2 the injected noise keeps the error high and unstable across rounds. This is consistent with the trade-off in Table 7 and explains why tight budgets sacrifice forecasting accuracy while, as noted above, scheduling latency remains stable because the load index still uses live state.

### Scalability

Table 8 maps from 50 to 500 nodes in the federation. The latency improves, not getting worse but better, from 151.8ms to 121.5ms, as more capacity carries the same proportion of load, and per run wall-clock increases in roughly linear fashion (15 to 81 s). The overhead of communication increases slightly as the number of nodes increases, from 0.38% at 50 nodes to 3.71% at 500 nodes, which is still acceptable. FPLB thus remains a small model, yielding benefits even at the size explored; it is never impractical compared to a purely local predictive model in the range explored.

**Table 8:** Scaling with federation size (arrival rate = 8 tasks/s).

Fog nodes	Latency (ms)	Comm. (%)	Wall-clock (s)
50	151.8	0.38	15.4
200	125.3	1.52	39.6
500	121.5	3.71	81.4

### Robustness to partial participation

Because of node failures, intermittent connectivity, and stragglers, per-round dropout is injected during fog deployments, where a percentage of nodes are prevented from sending updates (Table 9). There is no change for latency in the range of 0% to 30% dropout (149.2 ms) and no significant variation in prediction error across the range (0.26 MAE). Weighted FedAvg averages over the nodes that report and since many nodes observe the shared workload structure, they are not significantly impacted if a subset of them is missing in a round of the algorithm. As a result, FPLB gracefully breaks down when part of its participation is assumed away by the formal assumption of full-participation FedAvg.

**Table 9:** Robustness to per-round node dropout (arrival rate = 8 tasks/s).

Node dropout	Latency (ms)	Pred. MAE
0%	149.2 ± 0.0	0.26
10%	149.2 ± 0.0	0.26
20%	149.2 ± 0.0	0.26
30%	149.2 ± 0.0	0.26

### Sensitivity

The principal hyperparameters are varied in Table 10. A window of $$\:W\:=\:10$$ is adequate for the nodes with a limited amount of resources, providing that the prediction error is minimized (0.27 MAE), the latter being a bit worse, with $$\:W\:=\:20$$ giving no significant improvement. The prediction weight γ has an impact: Prediction has a contribution of about 2.6% when $$\:\gamma\:\:=\:0.25$$, and increases to 153.7 ms when $$\:\gamma\:\:=\:0$$ (no forecasting). The sensitivity of the offloading threshold $$\:\tau\:$$ is low for $$\:\tau\:$$ in [0.6, 0.9]$. The weights ($$\:\alpha\:,\beta\:,\gamma\:$$) were robust within the range of ± 0.1 of the weights chosen.

**Table 10:** Sensitivity to lookback * W*, prediction weight γ, and threshold τ (arrival rate = 8 tasks/s).

Setting	Latency (ms)	Pred. MAE
* W* = 5	150.2	0.31
* W* = 10	149.8	0.27
* W* = 20	149.7	0.32
$\:\gamma\:\:$= 0.0	153.7	-
$\:\gamma\:$ = 0.25	149.8	-
$\:\gamma\:$ = 0.5	149.7	-
τ = 0.6/0.75/0.9	149.8/149.8/149.8	-

### Network-delay term in the load index

Table 12 tests whether the excluded network-delay term $\:N_i$ should enter the load index. Including it slightly increases latency (149.2 → 151.3 ms) with a marginal utilization change, confirming that the queueing term already captures delay indirectly and that adding $\:N_i$ over-weights slow-uplink nodes. The omission is therefore justified empirically, not merely by convention.

### Resistance to membership inference

To test whether the shared model leaks which data a node used in training, a loss-based membership-inference attack was conducted. For the trained global model, the attacker compares its prediction error on member sequences (used in training) against non-member sequences drawn from the same generative process but never seen; a lower error is taken as evidence of membership, and attack success is measured by the area under the ROC curve (AUC), where 0.5 is random guessing. As Table 13 shows, the attack operates at chance across every budget, AUC ranges only from 0.49 to 0.50, and the member and non-member errors are statistically indistinguishable. Federated averaging over the 50 nodes, together with the shared temporal structure of the workload, prevents the model from memorizing any individual node’s trace, so membership is not recoverable even without differential privacy, and adding noise preserves this. Taken together with the privacy-utility sweep of Sect. 4.5, this shows that FPLB both quantifies the cost of a given budget and withstands a concrete privacy attack Table 13.

**Table 11:** Summarizes the evaluation scenarios covered, distinguishing those addressed in this work from those left as future work.

Scenario	Where	Covered
Varying workload intensity	Section 4.3, Table 5	yes
Heterogeneous CPU and network delay	Section 4.1	yes
Privacy budgets (ε sweep)	Section 4.5, Table 7	yes
Federation size 50–500 nodes	Section 4.6, Table 8	yes
Partial participation/node failure	Section 4.7, Table 9	yes
Non-IID workload (shared + local)	Section 4.1, 4.4	yes
Hyperparameter sensitivity	Section 4.8, Table 10	yes
Membership-inference attack	Section 4.10, Table 13	yes
Physical testbed deployment	Section 5	future work
Malicious clients/model poisoning	Section 5	future work

**Table 12:** Load index with and without the network-delay term (arrival rate = 8 tasks/s).

Load index	Latency (ms)	Util. (%)
without N_i_ (proposed)	149.2 ± 0.0	65.5
with N_i_	151.3 ± 0.1	67.4

**Table 13:** Loss-based membership-inference attack. AUC near 0.5 means the attack is no better than chance.

ε	Noise σ	Error (member)	Error (non-member)	Attack AUC
∞ (no DP)	0.00	0.110	0.109	0.492
3.2	5.06	8.028	8.037	0.499
1.0	15.50	23.707	23.568	0.497
0.5	30.68	47.182	46.618	0.496

## Discussion

FPLB is not the best method under every condition: at low load the overhead of prediction and aggregation outweighs its benefit and DQN is comparable, and least-loaded achieves marginally higher utilization by concentrating work. The structure of the framework becomes apparent under moderate-to-high load where the proactive placement of events does not trigger cascades of congestion and is predictable and stable under substantially lower latency variance compared to other frameworks, which is relevant for latency critical applications that pay attention to the tail behaviour as well as the mean behaviour. The following properties should be used as a guide for deployment: deployments with a consistently light load would not benefit from the added complexity of fog systems; deployments with a peak heavy or bursty load are best.

Federation has the advantage of being relatively cheap on cost compared to a central predictive alternative. The centralized scheduler needs to send per-node monitoring traces to a coordinator whose cost is proportional to the monitoring horizon, while FPLB only sends a fixed-size model (≈ 35 K parameters) once a round. This ensures that communication remains at 0.4% of traffic when there are 50 nodes and at 3.7% when there are 500 nodes, and that the raw data is stored on the node. At this small overhead, the measured latency improvement over the strongest learned baseline (DQN at the default load, up to 2.9% at high load) is real, but moderate. The better practical arguments are that it has a lower variance, gracefully handles the failure of nodes and privacy conditions, and avoids raw-trace sharing altogether. A centralized DQN would be a sensible and more straightforward option if the deployment prioritizes neither privacy nor predictability, and that’s exactly what it says on the tin.

Several limitations remain. Real testbeds introduce network instability and hardware effects that are abstracted by the evaluation in simulation. Aggregation is plain weighted FedAvg that doesn’t use client selection, compression, personalization, or asynchrony^24,25^ and as such is a result that may seem robust, but only directions for methodological improvements to solve the problems. The privacy analysis reports the utility cost of a given budget, a graceful-degradation property, and a membership-inference attack that performs no better than chance (Sect. 4.10); a complementary model-inversion analysis remains a natural next step. Finally, the load-index weights are global, and an adaptive per-node or time-varying scheme is a promising extension the sensitivity analysis motivates.

## Conclusion

This paper presented FPLB, which couples federated LSTM forecasting with a predictive load index and ($\:\epsilon\:,\:\delta\:$)-differential privacy for online fog load balancing. Under moderate-to-high load it attains the lowest and most predictable latency among the methods compared, significantly below DQN and a federated-DQN control (*p* < 0.01), with the margin widening as load grows and communication overhead held to a few percent even at 500 nodes. Ablation attributes the gain to prediction, federation improves prediction generalization, and the framework is robust to 30% node dropout and degrades gracefully as the privacy budget tightens. Its advantage is workload-dependent, so it is best suited to dynamic or peak-heavy deployments. Future work will pursue asynchronous and personalized aggregation, formal privacy-attack evaluation, adaptive load-index weighting, and validation on a physical fog testbed.

## Data Availability

The data supporting this study are not derived from third-party datasets. All data are synthetic and generated at runtime by the simulation code released with this article. The complete configuration -number of nodes, workload parameters, model hyperparameters, and random seeds - is specified in the manuscript and encoded in the released repository, so that every table and figure can be regenerated exactly by running the provided scripts. There are no access restrictions. The archive that reproduces all datasets and results is available at the repository cited in the Code Availability statement. The custom code implementing the FPLB framework, all baseline schedulers, the federated LSTM predictor with the differential-privacy accountant, and the full experiment suite is publicly available on GitHub at https://github.com/aartisharma1988/fplb. The implementation is in Python (NumPy, SciPy, PyTorch); the repository includes the exact configuration and seeds needed to reproduce every result reported here and a README describing how to run each experiment. No access restrictions apply.

## Code availability

The custom code implementing the FPLB framework, all baseline schedulers, the federated LSTM predictor with the differential-privacy accountant, and the full experiment suite is publicly available on GitHub at https://github.com/aartisharma1988/fplb. The implementation is in Python (NumPy, SciPy, PyTorch); the repository includes the exact configuration and seeds needed to reproduce every result reported here and a README describing how to run each experiment. No access restrictions apply.
